# New Insights in the Pathogenesis of Multiple Sclerosis—Role of Acrolein in Neuronal and Myelin Damage

**DOI:** 10.3390/ijms141020037

**Published:** 2013-10-09

**Authors:** Melissa Tully, Riyi Shi

**Affiliations:** 1 Weldon School of Biomedical Engineering, Purdue University, West Lafayette, IN 47907, USA; E-Mail: mtully@iupui.edu; 2 Indiana University School of Medicine, Indianapolis, IN 46202, USA; 3 Department of Basic Medical Sciences, College of Veterinary Medicine, Purdue University, West Lafayette, IN 47907, USA

**Keywords:** multiple sclerosis, acrolein, oxidative stress, autoimmune, neurodegeneration, demyelination, axonal injury, EAE, hydralazine

## Abstract

Multiple sclerosis (MS) is an autoimmune disease of the central nervous system (CNS) characterized by an inappropriate inflammatory reaction resulting in widespread myelin injury along white matter tracts. Neurological impairment as a result of the disease can be attributed to immune-mediated injury to myelin, axons and mitochondria, but the molecular mechanisms underlying the neuropathy remain incompletely understood. Incomplete mechanistic knowledge hinders the development of therapies capable of alleviating symptoms and slowing disease progression in the long-term. Recently, oxidative stress has been implicated as a key component of neural tissue damage prompting investigation of reactive oxygen species (ROS) scavengers as a potential therapeutic option. Despite the establishment of oxidative stress as a crucial process in MS development and progression, ROS scavengers have had limited success in animal studies which has prompted pursuit of an alternative target capable of curtailing oxidative stress. Acrolein, a toxic β-unsaturated aldehyde capable of initiating and perpetuating oxidative stress, has been suggested as a viable point of intervention to guide the development of new treatments. Sequestering acrolein using an FDA-approved compound, hydralazine, offers neuroprotection resulting in dampened symptom severity and slowed disease progression in experimental autoimmune encephalomyelitis (EAE) mice. These results provide promise for therapeutic development, indicating the possible utility of neutralizing acrolein to preserve and improve neurological function in MS patients.

## 1. Introduction

Multiple sclerosis (MS) is an autoimmune demyelinating neuropathy with a variety of endogenous and exogenous factors that are believed to influence disease risk and clinical course. Many reviews have focused on assessing patient susceptibility to MS, most notably relationships with gender, geography and genetics, which have demonstrated strong correlations in clinical cohort studies. However, to date, the molecular mechanisms underlying known pathogenic risk factors have yet to be established and, consequently, MS has remained largely refractory to available FDA-approved treatments. Marked progress in animal studies has enhanced understanding of pathological mechanisms allowing identification of novel therapeutic targets to improve quality and effectiveness of treatment regimens. Demonstrations of involvement of oxidative stress have led to attempts to sequester reactive oxygen species (ROS) for therapeutic intervention; however, to date, targeting solely ROS has had marginal success. This review summarizes current knowledge of MS pathogenesis focusing particularly on the role of acrolein, a toxic aldehyde and key factor in oxidative stress, in attempt to highlight the potential use of acrolein as a therapeutic target and a biomarker for MS diagnosis and disease progression.

## 2. Pathogenesis

Multiple sclerosis can be categorized into two main subtypes: relapsing remitting (RR) and primary progressive (PP), which differ in average age of onset, time course, and initial presentation [1]. RRMS, responsible for 85% of cases in predominantly younger patients, is characterized by one to two transient CNS attacks per year with either partial or complete symptomatic resolution. At an average age of 42 years, RRMS patients often transition to secondary progressive (SP) MS, a chronic phase characterized by attacks without recovery. Permanent neurological impairments of SPMS eventually provoke progressive physical deterioration of the patient [2,3]. The remaining 15% of cases typically present in older patients and are classified as PPMS [4]. PPMS shares many characteristics with SPMS, except that it is not preceded by RRMS; PPMS patients, even in initial stages of the disease, do not experience physical recovery following an attack.

Although the exact molecular mechanisms remain to be elucidated, the general consensus is that the clinical features of MS are the result of a triad of neural tissue injury mechanisms: inflammation, demyelination, and axonal damage [2,5]. Autoreactive myelin-specific T-lymphocytes are considered the main inflammatory culprits behind nervous system assault and initiation of disease processes [5–8]. Presumed to be activated by molecular mimicry, these lymphocytes initiate a cascade of subsequent events such as blood-brain-barrier disruption, microglial activation, excitotoxicity, plaque development, and ultimately neurodegeneration and microglial scarring. Furthermore, evidence of inflammation is apparent in biopsied plaques comprised of lymphocytes and macrophages, and in the blood and CSF which include myelin reactive T-lymphocytes [9–11]. Microglia contribute to the inflammatory atmosphere instigated by the T-cells by releasing proteolytic enzymes, cytokines, oxidative products, and free radicals all of which exhibit toxicity toward oligodendrocytes and myelin [1,3]. Inflammation elicits mitochondrial disruption, demyelination and axonal assault, the processes behind transient and permanent neurological impairment and conduction failure. In addition to myelin damage, axonal loss has also been recognized to contribute to the long term functional deficits and progression of disease [4,7,12,13]. Ultimately, compromise of axons, left vulnerable by loss of myelin sheath, is believed to elicit neuronal degeneration, cerebral atrophy, and permanent loss of function all of which are characteristic of late stage PPMS and SPMS.

Traditional anti-inflammatory therapies have so far demonstrated short term effects in reducing relapse rates but little if any benefit in slowing disease progression [5,14]. Development of a novel and more effective treatment strategy necessitates additional study of the underlying pathogenic mechanisms. Establishment of well-defined links between the observed inflammatory reactions, demyelination, and axonal damage may enable identification of more suitable pharmacologic targets.

## 3. Oxidative Stress

Current studies have suggested that the pathogenic traits of MS collectively implicate oxidative stress as a crucial factor in initiating and perpetuating mechanisms responsible for the characteristic progressive neurological impairment [15–18]. Processes responsible for generation of reactive oxygen species and lipid peroxidation, hallmarks of oxidative stress, have been studied in attempt to develop therapies that can slow, halt, or even reverse chemically-mediated CNS damage. Recent efforts have focused on targeting ROS by applying pharmaceutical agents to curtail oxidative stress and suppress free radicals, likely instigators of demyelination and subsequent axonal degeneration. Unfortunately, these efforts have had minimal effect *in vivo* which is thought to be attributed to their short half-life, on the order of milliseconds, and degree of instability of ROS [5,14,18]. Limited response to application of ROS scavengers has led to the pursuit of a stable compound associated with oxidative stress to serve as a target for therapeutic intervention. Acrolein (2-propenal), a more stable compound capable of perpetuating oxidative stress by both initiating and bolstering lipid peroxidation and ROS generation, has been linked to a plethora of diseases [19–23]. Researchers have provided substantial data illustrating the detrimental nature of acrolein, particularly in the nervous system.

## 4. Acrolein

Acrolein, a β-unsaturated aldehyde, is a powerful toxin that has been shown to damage proteins, lipids, and DNA, generate free radicals, and activate cascades of noxious enzymes [17,24,25]. Acrolein has a variety of endogenous and exogenous sources. As a pollutant, acrolein is emitted during various manufacturing processes, burning cigarettes, exhaust from combustion engines, and vapors of overheated cooking oil [24]. Acrolein is also generated endogenously through the oxidation of various compounds, achieving pathological concentrations in conditions such as trauma, stress, and aging [26]. Both external and internal sources can elevate acrolein concentrations to potentially damaging levels within the body [17,27–31]. As both a product and catalyst of lipid peroxidation, acrolein induces a vicious cycle of oxidative stress, dramatically amplifying its effects [24,32]. Furthermore, acrolein remains active in the body much longer than commonly studied oxidative species; most oxygen radicals decay within fractions of a second, while acrolein persists for seven to ten days [33]. Compared to other aldehydes associated with oxidative stress such as 4-hydroxynonenal, acrolein is generated at 40 times greater concentration and is 100 times more reactive [24,32,33]. Therefore, acrolein is the most reactive and abundant aldehyde. This evidence strongly suggests that acrolein is a key factor in perpetuating oxidative stress and has the potential to serve as a viable therapeutic target.

Studies have demonstrated that acrolein is capable of directly damaging nucleic acids present in DNA, amino acids and lipids, all crucial components of myelin sheath and the lipid-rich neuronal membrane. Through reaction with a wide variety of proteins and GSH, acrolein adducts have also exhibited significant reactivity and a longer half-life than that of free acrolein. Furthermore, a recent study has demonstrated that sequestering acrolein in an animal model of MS, through the use of acrolein scavengers, promotes functional recovery and decreases incidence of demyelination [27]. As evidenced in the aforementioned study, acrolein is likely a key mediator in sustaining or even further aggravating the inflammatory environment presumably liable for functional deterioration in MS and an assortment of other diseases attributed to oxidative stress [27]. An increase in acrolein-lysine adduct level was observed in EAE mice concordantly with the presence of behavioral deficit. In addition, treatment with an acrolein scavenger, hydralazine, reduced demyelination, slowed disease progression and improved behavioral score [27]. These studies further implicate acrolein as a pathologic factor in MS by demonstrating the attenuation of behavioral deficit and myelin damage in response to neutralizing acrolein with hydralazine.

## 5. Demyelination

CNS demyelination, the hallmark of MS development and progression, is responsible for the characteristic transient functional impairment seen clinically in RRMS [5,14]. Nerve conduction is adversely affected by myelin loss due to increased energy required for action potential propagation. Ultimately, increased energy expenditure depletes ATP and if myelin integrity is not restored, impulse conduction is halted. Previous evidence has indicated that myelin also plays an integral role in localizing expression of voltage-gated sodium (VGNa) to the nodes of Ranvier and voltage-gated potassium (VGK) channels to the juxtaparanodal region beneath myelin sheath. Decompaction or degradation of myelin results in the aberrant channel expression in which both VGNa and VGK channels are distributed along the entire length of the axon. VGNa channel redistribution is considered a compensatory mechanism permitting temporary preservation of conduction despite myelin breakdown and compromised nodes of Ranvier. However, VGNa channel rearrangement ultimately becomes ineffective, leading to an ionic imbalance and extracellular calcium influx, which is further exacerbated by VGK channel migration from the juxtaparanodal region. In addition, aberrant VGK expression resulting from acrolein-mediated myelin retraction can cause inappropriate outward movement of potassium ions and the failure of action potential generation at nodal region [34–36].

It is known that acrolein can directly damage myelin, likely through reaction with the protein and lipid components of the myelin sheath [27,35,37]. Specifically, when isolated *ex vivo* spinal cord was exposed to acrolein, significant alterations in myelin structure were observed including myelin retraction from the nodes of Ranvier and myelin splitting or decompaction [35]. Consequently, myelin retraction exposed VGK in the juxtaparanodal region, a well-established mechanism that culminates in conduction failure. Increased expression of VGK was reported to extend beyond the juxtaparanodal region into the nodes of Ranvier when exposed to acrolein for up to12 h [35,38]. It is hypothesized that localized expression of VGK is dictated by axoglial septate junctions. When acrolein-mediated damage at the junction damages anchoring proteins, channel movement is no longer inhibited facilitating aberrant VGK expression within the nodal region. Evidence supporting the notion of acrolein-mediated damage of axoglial septate junctions is that Caspr, an anchoring protein expressed in the paranodal region of healthy neurons, is delocalized following acrolein exposure [35,36]. In addition to directly reacting with myelin constituents, acrolein exposure activates calpain, an enzyme involved in microtubule degradation, enhancing the already toxic environment [39–41].

Though demyelination and membrane damage were previously considered separate disease processes, they now appear to be intertwined; both have an impact on ionic balance and channel distribution essential for usual axonal conduction [42–45]. Disruption of these two components by acrolein is likely to be at least partly responsible for the symptoms and functional deterioration observed in MS patients. This hypothesis is supported by the fact that acrolein is capable of damaging both myelin and axons directly and indirectly. Myelin retraction and loss alters the activity and induces aberrant expression of many ion channels resulting in conduction failure and functional decline. Concordantly, axonal damage also leads to conduction failure and neuronal degeneration, and, in addition, permanent neurological dysfunction characteristic of PP- and SP-MS. Therefore, it is reasonable to suggest the role of acrolein in dual pathological processes in MS.

## 6. Axonal Injury

The importance of axonal degeneration as a pathologic mechanism in MS has been demonstrated by post-mortem studies that directly examined lesions in MS patient brain tissue using markers for axonal damage and myelin disruption. These studies observed a great number of transected axons, implicating axonal injury as a critical feature of neurodegeneration likely responsible for permanent neurological deficit often seen in late stages of MS [13,46]. This idea is further supported by the evidence that axonal damage provoked by an inflammatory environment results in conduction failure and subsequent symptom development in MS animal models and clinical cases [2,47]. Furthermore, axonal injury likely represents permanent neuron impairment, and ultimately degeneration, serving as a likely explanation for absence of functional recovery in PP- and SP-MS [13,46]. In instances of axonal damage in demyelinated axons, atypical expression of glutamate receptors, sodium channels, and voltage-gated calcium channels has been reported [17,25]. These results indicate that axonal injury coupled with altered channel expression, resulting from myelin decompaction, may increase intracellular calcium levels, all contributing to the degeneration of axons.

As mentioned, acrolein is capable of inflicting axonal damage directly. This is likely because the axonal membrane, like myelin, is lipid rich and therefore equally susceptible to oxidative insult—especially acrolein attack. On the other hand, demyelination not only impairs conduction through loss of internodes and aberrant channel expression, but also leaves the axonal membrane exposed and vulnerable to injury by inflammatory mediators released by activated glial cells, and acrolein. Taken together, acrolein can damage axons through direct and indirect mechanisms.

Shi and colleagues have demonstrated that micromolar concentrations of acrolein can disrupt the lipid rich cellular membrane, and, moreover, elevated acrolein levels are accompanied by observable increase of axonal permeability and loss of compound action potential (CAP) conduction [17,30,48]. Introduction of a known acrolein scavenger, hydralazine, in an *in vivo* or *in vitro* model was able to alleviate axonal damage. These studies have provided compelling evidence that acrolein is indeed capable of inflicting axonal damage and likely plays a significant role in axonal loss in an MS animal model [27].

## 7. Mitochondrial Dysfunction

Mitochondrial impairment is an important mechanism underlying observed deficits characteristic of MS causing deficient energy production provoking cell death. Demyelination and axonal membrane compromise lead to calcium influx, with consequent activation of cellular pathways provoking harmful mediators that directly attack mitochondria [14]. The hostile inflammatory environment and membrane deterioration associated with oxidative stress suggest that mitochondria may also be vulnerable to the effects of acrolein and ROS more than other cellular structures. Mitochondria provide energy to the cell in the form of ATP to promote cell function, growth and survival. The process by which they are able to generate energy, results in the non-pathological production of ROS by the electron transport chain. Mitochondrial dysfunction impairs the ability of neurons to combat oxidative stress and allows the release of endogenous ROS produced by the mitochondria. Thus, in disease states, mitochondria are particularly susceptible to the damaging effects of oxidative compounds and furthermore can exacerbate oxidative stress and promote cell death and subsequent axonal loss [49,50].

Acrolein has been shown to cause dysfunction of mitochondrial respiration in heart, spinal cord and brain tissues [25,51–53]. Recently, evidence has been obtained revealing that acrolein may be able to directly damage mitochondria. Specifically, it has been demonstrated that isolated mitochondria, when exposed to acrolein, generate ROS and deplete GSH [25]. Reduction of GSH by acrolein renders brain mitochondria highly susceptible to oxidative stress due to their low levels of catalase [25]. The notion of direct attack of mitochondria by acrolein was further strengthened by the fact that acrolein impairs adenine nucleotide translocase (ANT) and consequently, the electron transport chain. It was suggested that acrolein can directly inhibit ANT, likely through binding cysteine residues, ultimately diminishing local energy production, perpetuating oxidative stress, and severely impairing mitochondria function [25,54,55]. This hypothesis is supported by evidence that dysfunction of the electron transport chain can be facilitated by inhibition of ANT and depletion of glutathione by increased mitochondrial ROS production [56–58]. If these mechanisms are indeed valid, acrolein would be capable of both inhibition of ANT and stimulation of ROS production within mitochondria [25].

## 8. Acrolein Scavenging—A Novel Treatment Strategy for MS

Since acrolein has been implicated as a factor capable of instigating and perpetuating the main pathogenic mechanisms underlying MS, it is a logical target for pharmacologic therapeutic targeting. Compounds capable of binding and neutralizing acrolein have shown great promise by alleviating elevated acrolein levels and attenuating functional decline in EAE mice [42]. Some drugs currently being examined, such as hydralazine, an FDA-approved anti-hypertensive, are show to be effective acrolein scavengers in both *in vitro* and *in vivo* studies at safe doses [27,48,59]. When administered to EAE mice in doses that are substantially less than those approved clinically for antihypertensive use, hydralazine is capable of sequestering acrolein, mitigating behavioral decline and alleviating demyelination seen in histological preparations [27]. Similar beneficial effects were also seen in SCI where acrolein has been implicated. Considering the ability to scavenge acrolein and its FDA-approval, we speculate that the hydralazine-based anti-acrolein strategy could be developed into a new MS therapy that can be rapidly translated into the clinic.

Ideally, application of acrolein-scavenging drugs would ease motor deficit through reduction of acrolein-associated oxidative stress and subsequent membrane, myelin and mitochondrial insult and ultimately be translated to the clinic as a viable treatment available for MS patients. The main advantage of this novel approach includes a clear, identifiable, molecular target for treatment, established methods of monitoring acrolein levels for potential diagnosis, treatment selection and evaluation. Beyond amplifying oxidative stress, acrolein synergistically exacerbates other parallel pathologies that are potentially involved in MS pathology, such as ischemia, inflammation, and excitotoxicity [60–65]. Therefore, once established anti-acrolein therapies could also be paired with other interventions to maximize treatment effects and greatly enhance the quality of life for MS patients.

## 9. Concluding Remarks

There is strong evidence to support that oxidative stress plays a role in course of MS and symptom development. Acrolein appears to be the key factor perpetuating oxidative stress. Recent studies have ascertained that ROS and acrolein are at least partially responsible for the three hallmark mechanisms underlying the disease: axonal membrane damage, demyelination, and mitochondrial dysfunction. More specifically, acrolein is capable of damaging the membrane, myelin, and mitochondria in the nervous system. Elevated levels of acrolein have been detected in CNS tissue in EAE mice when symptoms peak. In addition, the acrolein scavenger, hydralazine, has been shown to reduce symptom severity and slow disease progression through a different mechanism than the currently approved treatments. This information provides compelling evidence supporting the role of acrolein in functional deterioration and characteristic mechanisms associated with MS. Therefore, acrolein may contribute to the pathogenesis of the disease and has the potential to serve as a novel target for the effective use of acrolein scavengers for long-term symptomatic improvement in MS patients. Furthermore, this strategy may also be efficacious in the treatment of other diseases associated with oxidative stress such as cancer, diabetes, atherosclerosis, SCI, and other neurodegenerative disorders.
